# Characterizing Sex Ratios of American Eels ( *Anguilla rostrata* ) in Louisiana

**DOI:** 10.17912/micropub.biology.001256

**Published:** 2024-07-12

**Authors:** Valeria Faria, Robby Maxwell, Sean Kinney, Amber Hale

**Affiliations:** 1 Department of Biology, McNeese State University; 2 Louisiana Department of Wildlife and Fisheries

## Abstract

The American eel ( *Anguilla rostrata)* inhabits Louisiana waterways; however, little is known about their life history, population abundance, or behavior. Eels under 400 mm require histologic evaluation to determine sex. We have processed eel gonad samples from 40 sampling locations across Louisiana, as well as across size categories to aid in establishing a sex determination protocol. One hundred and eighteen (118) eel samples have been histologically analyzed to date. The histologic data compliments morphometric, location, and ageing data collected by the Louisiana Department of Wildlife and Fisheries to build an initial understanding of the biological characteristics of American eels in Louisiana.

**Figure 1. American eels in Louisiana:**
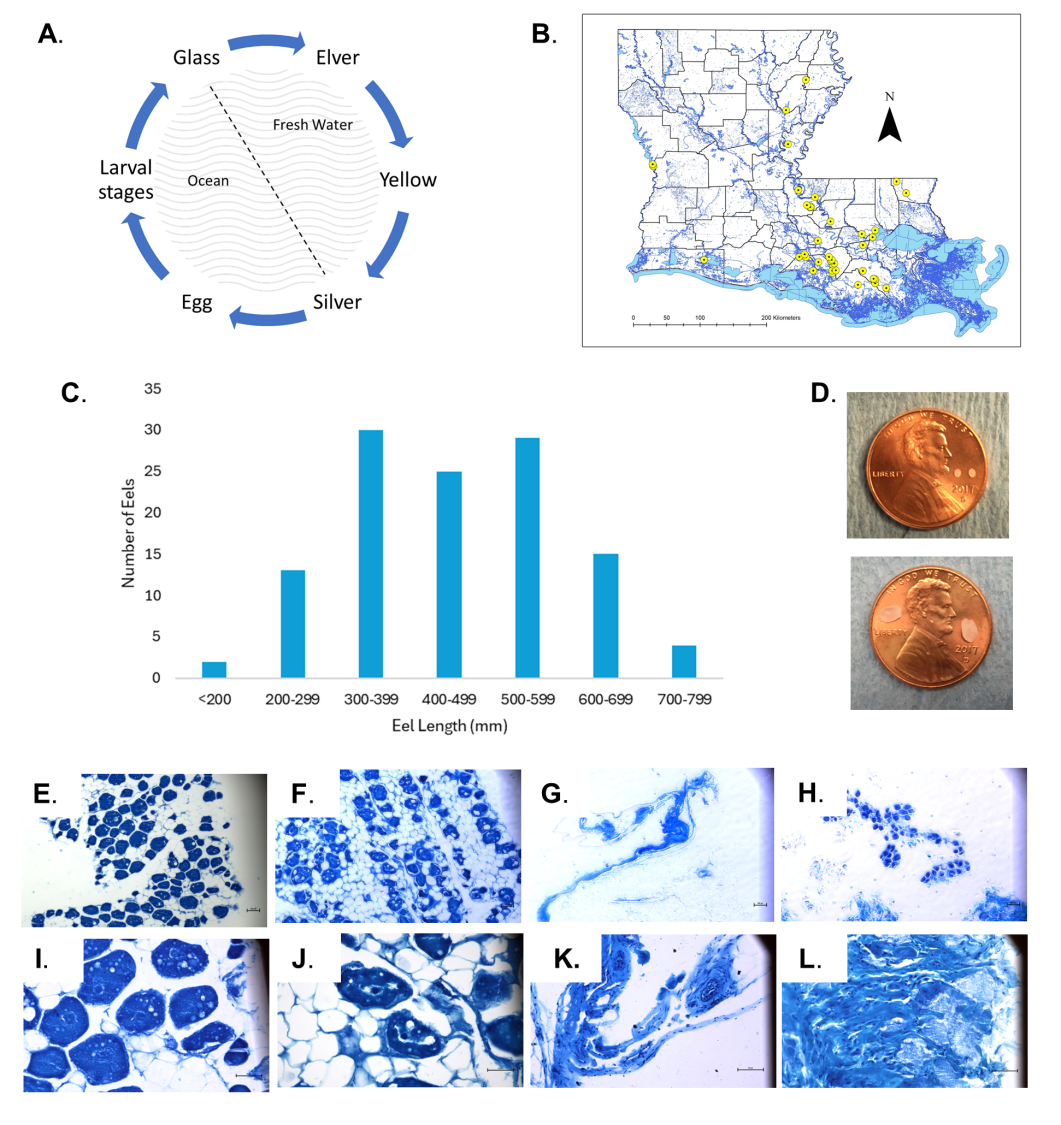
Panel A. Eel life cycle. American eels spawn in the Sargasso Sea. Once in the larval stage, called *leptocephali* , they drift with the ocean currents to the coasts. Glass eels enter the brackish, coastal waterways then, as the eels continue up the waterways into fresh water, they metamorphose into elver then yellow stages. Yellow eels eventually mature into the silver eel stage, at which point they return to the oceanic spawning site to spawn and die. Panel B. Louisiana map depicting locations of eel sampling sites. American eels were captured from 40 sites across Louisiana waterways noted in the figure. Sites varied in distance from the coast as well as habitat. Eels were primarily captured as electrofishing bycatch. Panel C. Eel Length Distribution Eels were measured upon collection. Eels were grouped based on length at 100 mm intervals. The histogram depicts the distribution of frequencies. Seventy-one percent of eels collected measured 300-599 mm. Panel D. Otolith Examples Eel ages were determined via otolith analysis. The otoliths are photographed on top of pennies for scale. The otoliths in the upper image are from a 182 mm eel, and the lower image is of otoliths taken from a 683 mm eel. Panels E.-F., I.-J. Representative large eel specimens. All large eels (>400mm) were found to be histologically female and in the yellow eel stage. The figures depict eel ovaries containing various stages of oocyte development and adipose tissue. **Panel E., I.** Gonadal tissue from eel 42, a large female (731 mm in length, 958 g weight, 11 years of age). Some oocytes with nuclei are visible, some cortical alveoli (lipid droplets) can be seen. Several adipocytes surrounding the gonadal tissue are also visible. We find that additional adipose tissue deposits are typical of aged females. **Panel F., J.** Gonadal tissue from eel 87, a large female (794 mm in length, 1211 g weight, 9 years of age). Similar structures and appearance of eel 42. Panels E.-F., 100X total magnification (TM), Panels I.- J., 400X TM Panels G., H., K., L. Representative small eel specimens Samples from smaller specimens obtained for this investigation largely consist of loose connective tissue and smooth muscle. These eels were not yet at the yellow eel stage (likely at the elver eel stage) based on length and age. While one specimen appeared to have early signs of ovarian development, none of the smaller eels were mature enough to have fully developed oocytes, as expected. **Panel G., K.** Gonadal tissue from eel 71, a small eel with an undifferentiated gonad (214 mm, 15 g, 2 years of age). Smooth muscle, vasculature, and loose connective tissue comprise the majority of the sample. **Panel H., L.** Gonadal tissue from eel 92, a small eel with a female gonad (247 mm in length, 26 g weight, 2 years of age). Eel 92 illustrates the transition from an undifferentiated gonad containing mostly loose connective tissue to a recognizable ovary. Immature oocytes can be seen, though the oocytes, as well as the overall tissue are comparatively small. Panels G.-H., 100X TM, Panels K.- L., 400X TM

## Description

American eels ( *Anguilla rostrata)* are a long-lived, catadromous species. American eels spawn in the Sargasso Sea (panmictic population) from where the larval stages, called *leptocephali* , drift to the coasts of the Atlantic Ocean, Gulf of Mexico, and Caribbean Sea carried by the ocean currents (Ulmo-Diaz et al. 2023) . The exact spawning location in the Sargasso Sea, as well as other, fundamental natural history knowledge continue to be researched as eels are tracked across their range (Hanel et al. 2022) . It is known that American eels enter the coastal waterways in the glass stage and migrate inland whilst they grow through several morphologic stages (elver, yellow). Once they are prepared to reproduce, the eels morph into the silver stage, and migrate back to their spawning location (see eel life cycle, panel A). Furthermore, it has been shown that some eel populations are spatially stratified, where more inland populations are exclusively female and coastal populations are mixed, often with higher proportions of males (Tremblay et al. 2009).

Sexual differentiation in eels is largely environmentally determined. Though a genetic basis exists, as females have been observed to have a pair of heteromorphic chromosomes, environmental factors often override the chromosomal component (reviewed in Tesch 2003). As eels mature through the glass and elver stages, the undifferentiated gonad may pass through an intersex stage (Syrski organ) prior to differentiation, or an ovary may develop directly from the undifferentiated stage (Colombo and Grandi 1996) . In the early yellow stage of development, sex cannot be determined without histologic evaluation. Thus, the purpose of our study was to assess gonadal tissue histologically to determine sex.

Eels were collected at 40 sites in Louisiana, both coastal and inland (see Louisiana map, panel B), and 118 eels gonads were analyzed histologically. The length of the eels analyzed ranged from a minimum of 182 mm to a maximum of 794 mm. The length distribution is reported in panel C. Seventy-three of the sampled eels were 400 mm or greater in length (range 400-794 mm) (Maxwell and Kinny 2021) and all of these were found to be female via histological analysis. The age range of this size class was 2-14 years old (no otoliths/ageing data were recovered from one sample). Only one eel determined to be 2 years old was found to be >400 mm in length (452 mm). Refer to panel D for examples of collected otoliths.

Forty-five eels that measured less than 400 mm were assessed. They ranged in age from 2-16 years and their lengths ranged from 182-394 mm. Twelve of these smaller eels were found to be 2 years of age or younger (26.7%). Five of the six smallest eels had undifferentiated gonads (length range 182-247 mm), supporting the hypotheses that the less developed eels would not have differentiated gonadal tissue. The remaining smallest eel had a differentiated, female gonad.

The eels with undifferentiated gonads did not contain any visible oocytes or spermatocytes. The elver (i.e. juvenile) eel gonads consisted of connective tissue, smooth muscle, and adipocytes (Panels G., H., K., L.). The eels that were at the yellow eel stage did display oocytes with nuclei (Panels E., F., I., J.). These larger eels also had more fatty tissue and volume of gonadal tissue present. In agreement with the published literature on other *Anguilla* species, all eels 400 mm or above that were analyzed were morphologically female.

We were surprised that we did not find any examples of the intersex gonad, as we had a relatively large sample size (n=118) collected over several seasons, and at various sampling locations. More surprisingly, we did not identify any male specimens. Other studies have observed ~35% females in the population, with no effect of salinity or density on sex ratios (Côté et al. 2015). It has been shown in longfinned eels ( *Anguilla reinhardtii* ) that males are more prevalent in tidal (coastal) locations (Walsh et al. 2004) , so it is possible that more coastal sampling, or a different sampling technique may yield greater numbers of males. In any case, the results presented are noteworthy and warrant further study.

*Anguilla rostrata* is an understudied species worldwide, and especially so on the Gulf coast. It is known that eel habitat preference is dependent on life stage, size, and/or sex (reviewed in Arai 2020). With so little known about this native Louisiana species, it is impossible to determine if they are threatened by development and waterway control, if their population is stable, or if it is declining. Further research is necessary to gain a better understanding of the status of the American eel.

## Methods

Sample Collection

The American eels were collected by LDWF personnel, mostly as electrofishing bycatch. Eels were frozen immediately after collection and maintained at -20°C for varying durations. The gonadal tissues were thawed with the whole eel, dissected out, fixed in 10% neutral buffered formalin overnight at room temperature then transferred to 70% ethanol until processed for histology.

Otolith Ageing

Otoliths were aged by the Louisiana Department of Wildlife and Fisheries Age and Growth Lab. Otoliths were embedded in bullet molds with the medial side down. Embedded otoliths were then sectioned on a low-speed wafering saw using two blades separated by a plastic spacer with a width of approximately 0.4mm. Under a stereomicroscope, the core was marked prior to sectioning. Sections were mounted on labeled slides with Loctite and covered with Shandon-Mount permanent mounting medium. Ages were estimated as a “continental age” beginning at January 1, meaning the age at which the eels left oceanic waters and moved inland (Maxwell and Kinny 2021) .

Histology

Samples were dehydrated via a graded ethanol series and paraffin embedded. Tissue blocks were cut to a thickness of 7 µm using a Leica manual rotary microtome and mounted on glass slides. Samples were stained with hematoxylin and eosin (H&E) and/or Wright’s stain and coverslipped with permanent mounting medium.

Microscopy

Slides were viewed and imaged using brightfield illumination. A Nikon 50i microscope and the companion NES Elements software were used to capture images.
